# Student Health Services at Historically Black Colleges and Universities and Predominantly Black Institutions in the United States

**DOI:** 10.1089/heq.2023.0219

**Published:** 2024-03-25

**Authors:** Susan D. Mueller, Melissa A. Sutherland, M. Katherine Hutchinson, Bing Si, Yu Ding, Somatra L. Connolly

**Affiliations:** ^1^ Tompkins Cortland Community College, Dryden, New York, USA.; ^2^ College of Nursing, University of Rhode Island, Kingston, Rhode Island, USA.; ^3^ Systems Science and Industrial Engineering, Binghamton University, Binghamton, New York, USA.; ^4^ Rhode Island Nursing Education Center (RINEC), College of Nursing, University of Rhode Island, Providence, Rhode Island, USA.

**Keywords:** HBCU, Predominantly Black Institutions, Minority Serving Institutions, health equity, campus health

## Abstract

**Introduction:**

Student health services are associated with improved health outcomes and academic success, particularly among under-resourced college populations. This study compared student health services at Historically Black Colleges and Universities (HBCUs) and Predominantly Black Institutions (PBIs) and identified factors associated with the availability of comprehensive health services (CHS).

**Methods:**

We conducted a secondary analysis of 2022 data from the Integrated Postsecondary Education Data System (IPEDS), the Minority Serving Institutions (MSIs) Directory, and the websites of HBCUs and PBIs (*n*=167). Bivariate and multivariate logistic regression analyses were undertaken to identify institutional variables associated with providing CHS. Institutional variables included college type (public vs. private), MSI category (HBCU vs. PBI), undergraduate enrollment, location, and proportion of Pell grant recipients.

**Results:**

Approximately 13% of HBCUs and 26% of PBIs offered no student health services; 65% of HBCUs and 39% of PBIs offered on-campus CHS with prescribing providers. Four-year HBCUs were five times more likely than 4-year PBIs to have CHS (*p*=0.014). Institutions with more Pell Grant recipients were less likely to offer CHS.

**Conclusions:**

Access to health care is an important social determinant of health, academic persistence, and achievement for college students. HBCUs were significantly more likely than PBIs to offer CHS. HBCUs are more likely than PBIs to have resources from federal funding, donors, and endowments that may support the development of student health centers and services. Increased funding for PBI health centers could improve access and promote health equity among the most vulnerable student populations.

## Introduction

Health equity, the ability of all people to have a fair opportunity to achieve wellness, is significantly influenced by access to health services.^1–3^ According to the “Right to Equitable Access to Available Resources for Health Model,” access to resources such as health services is essential to increasing health equity among marginalized populations and populations experiencing health disparities,^4^ which includes Black college students. Higher campus health center utilization has been noted among Black college students compared with White students,^5,6^ and utilization has been linked to positive student academic persistence,^7^ health-promoting behaviors,^5,8,9^ and chronic disease management.^6^

Students at Historically Black Colleges and Universities (HBCUs) and at Predominantly Black Institutions (PBIs) are more likely to experience basic need insecurities and financial strain than those at Predominantly White Institutions (PWIs), placing them at risk for physical and mental health conditions requiring services.^10–16^ Given this risk, access to health services is particularly important for students at HBCUs and PBIs.

## Literature Review

### HBCUs and PBIs: history and comparison

HBCUs and PBIs are Minority Serving Institutions (MSIs) that serve the Black student population.^17^ HBCUs were established before 1964 for the primary purpose of educating Black Americans.^17–19^ PBIs, which enroll at least 40% Black students and at least 50% Pell Grant eligible students,^20,21^ became an established MSI category in 2008.^5,22,23^ One key difference between these institutions is that HBCUs are mission-driven, while PBIs are identified/categorized using student demographics alone.^18,23–27^ Dedication to improving the circumstances of Black students is inherent in every aspect of HBCUs,^25,28–31^ while the commitment to holistic student support is not reflected in the same way at PBIs.^32^ This difference in institutional purpose informs many aspects of student life, including student services at HBCUs and PBIs.^18,23,29,32^

HBCUs and PBIs represent only 6% of accredited U.S. colleges and universities, but educate ∼18% of the Black college student population.^6,7^ Black students often choose HBCUs because of an increased sense of belonging, safety, and ability to have needs met.^28^ HBCUs and PBIs enroll a disproportionate number of low income and first generation students,^10,11,22,25,32^ two populations that often struggle with academic persistence and degree completion.^33,34^ Nationally, the 150% (6-year) completion rate for White students enrolled in 4-year institutions is 62–69%, compared to 40–43% for Black Students.^35^ The completion rate for Black students rises to 51.5–62.7% at PBIs and 61.8–66.7% at HBCUs.^34–36^

HBCUs are an essential mechanism for upward economic mobility in the Black population,^25,37^ and PBIs have demonstrated a commitment to the education of Black students within their local communities.^18,26^ Despite their role in educating and improving life course outcomes for Black college students,^6,38,39^ both HBCUs and PBIs are relatively underfunded compared to PWIs. HBCUs have historically received disproportionately low amounts of federal and state grant funding,^40,41^ lower gift amounts from private foundations,^40,42^ and have had less success with fundraising from alumni and other private donors than comparable PWIs.^37,40,42^ Lack of funding, which contributes to understaffing and inadequate physical facilities, interferes with the ability of Black-serving institutions to meet the holistic needs of their students.^40^

PBIs spend less per student than comparable PWIs,^5,17,18^ have smaller donor bases than HBCUs,^6^ and have underfunded endowments.^38^ Targeted funding is available for both HBCUs and PBIs through federal grant agencies^19,43^; however, there are fewer federal funding opportunities for health centers and health programs at PBIs than HBCUs.^19,43,44^ The United Negro College Fund (UNCF) provides funding avenues to increase opportunity for Black students in higher education^37^; including support for mental health centers at HBCUs,^45^ although there are no similar initiatives for general campus health centers.

### College health services

College populations experience the health inequities evident in the general population, with disparities^46,47^ noted in morbidity,^48^ depression and anxiety,^49^ and sexual health.^50^ Access to on-campus student health services is associated with improved health outcomes and academic persistence and can facilitate access to health care despite financial barriers (e.g., lack of parental contribution or access to parental health insurance),^12,51–55^ particularly among under-resourced groups. Student health centers fall under the umbrella of student services. Student perception of robust and supportive student services has been positively associated with both retention and degree completion.^31,52,53^ This is noteworthy among students at HBCUs and PBIs, who often have fewer financial resources than students at PWIs.^10,11,29,30^

Black Americans are disproportionately affected by persistent health inequities^8,56^ that are intertwined with access to health care, health promotion resources, and other determinants of health.^9,57^ Among racial and ethnic groups, Blacks have the highest poverty rates in the United States^58^; and groups considered poor or near-poor are more likely to be uninsured and lack health-related financial resources.^58^ Of HBCU students who receive emergency aid funds, 19% use those funds to cover medical bills.^11^ Black college students are disproportionately affected by poverty compared to White students,^59,60^ and ∼20%^61^ are uninsured. Black college students are more likely to utilize student health services compared with White students^62,63^ and are less likely to have a “usual place to go” for medical care.^47,58^

Campus health centers are a key facilitator of success among Black students.^17,54^ According to the American College Health Association (ACHA), the ideal student health center provides on-campus comprehensive health services (CHS), including access to prescribing providers at an institution-run clinic.^64,65^ Despite this recommendation, the quality and availability of student health services vary widely. While some colleges offer CHS staffed by prescribing providers,^64,66^ others provide no student health services, basic on-campus services provided by a nurse or emergency medical technician (EMT), and/or contract for off-campus or telehealth services.^60^ Differences in the availability and quality of student health services on college campuses may be due, at least in part, to financial constraints at individual institutions.^67^

Student health services are funded through several mechanisms, including insurance reimbursement, student fees, grants, loans, and institutional subsidies.^68,69^ HBCUs receive less funding than PWIs for both general programs and health services.^18,70–72^ Within Black-serving MSIs (HBCUs and PBIs), PBIs have fewer federal funding opportunities for health centers and health programs compared to HBCUs. This disparity in funding is a result of the HBCU Capital Financing Program, which offers noncompetitive subsidized loans for HBCUs to improve infrastructure in many areas, including student health services.^73^ For PBIs, neither U.S. Department of Education (DOE) formula grants^44^ nor competitive grants^43^ include or allow for discretionary funding of student health centers.

Despite the importance of student health centers in college students' health and academic success, there is limited literature about student health centers at HBCU and PBI institutions. The current study addressed this significant gap in the literature. The study aims were to: (1) examine and compare the on-campus student health services offered at 2- and 4-year HBCUs and (2) identify the institutional characteristics associated with the on-campus student health services offered at 4-year HBCUs and PBIs.

## Methods

### Procedures

This cross-sectional secondary analysis study utilized data from multiple sources, including the Integrated Postsecondary Education Data System (IPEDS),^74^ the Rutgers MSI Directory,^75^ and institution (university/college) websites. All data included in the dataset were publicly available through the IPEDS, the MSI Directory, or the university/college websites in 2022. No identifiers were included in the dataset and no human subjects participated; institutional review board approval was not required.

Institutional data were downloaded and included in the current study if the university/college was classified as either an HBCU or PBI located in 1 of the 50 U.S. states or the District of Columbia. A total of 167 institutions met the inclusion criteria. Data for percent of Black students, undergraduate enrollment, percent of Pell grant recipients, and National Center for Education Statistics (NCES)-defined setting were obtained by searching the common dataset for individual institutions.^76^ Information about student health services was obtained by reviewing the websites of the 167 institutions. Data were initially downloaded into Google Sheets and then exported to the R platform^77^ for data cleaning and statistical analyses.

### Dependent variable: college health services

A variable reflecting the student health services available on campus was created (Table 1).

**Table 1.** Distribution table of campus health care services by Minority Serving Institution type

	PBI, ***n*** (%)	HBCU, ***n*** (%)	***p*** ^a^	4-Year PBI, ***n*** (%)	4-year HBCU, ***n*** (%)	***p*** ^b^
Campus health services	71 (100)	96 (100)		23 (100)	85 (100)	
No health center	46 (64.79)	17 (17.71)	<0.001^c^	6 (26.09)	11 (12.94)	0.23
Staffed center with LPN, RN, or EMT^d^	8 (11.27)	16 (16.67)	0.45	5 (21.74)	11 (12.94)	0.47
Telehealth or contract	7 (9.86)	8 (8.33)	0.95	3 (13.04)	8 (9.41)	0.90
CHS	10 (14.08)	55 (57.29)	<0.001^d^	9 (39.13)	55 (64.71)	0.05^c^
	*p*<0.001^d^			*p*=0.16		

^a^ *p*-Value for the full sample.

^b^ *p*-Value for 4-year only.

^c^ *p*≤0.001.

^d^ *p*≤0.05.

CHS, comprehensive health services; EMT, emergency medical technician; HBCU, Historically Black College and University; LPN, licensed practical nurse; PBI, Predominantly Black Institution; RN, registered nurse.

Based on information available on institutional websites, universities/colleges were labeled and coded as: 0=no student health services available; 1=licensed practical nurse (LPN), registered nurse (RN), or EMT-run student health services; 2=student health services provided through third party contracts at either on- or off-campus clinics; or 3=on-campus CHS. CHS are clinics staffed by prescribing providers that provide care as defined by the Healthy Campus Framework model.^25^ A variable reflecting the student health services available was created for the descriptive analyses (Table 1). A dichotomous dummy variable was later created and coded as either CHS provided (1) or CHS not provided (0); the latter included none; LPN, RN, or EMT-run; or third-party health services.

### Independent variables

Institutional type was obtained from IPEDS^74^ and coded as 2-year (1), private 4-year (2), or public 4-year (3) and later recoded. MSI category was defined using DOE designations and reported by the Center for MSIs.^75^ Institutions were classified as either PBI (0) or HBCU (1). The categories PBI and HBCU were mutually exclusive. The percentage of students who self-identify as Black and percentage of Pell Grant Recipients were collected from information reported by individual colleges to the common dataset.^76^ Undergraduate enrollment was based upon the total number of undergraduates enrolled in 2021 as reported to the common dataset.^76^ Setting was defined using the NCES schema and coded as rural (0), urban (1), suburb (2), or town (3).^78^

### Statistical analysis

R software^77^ was used for data processing and analysis. Statistical significance was set at *p*≤0.05 for all analyses. Bivariate analysis was conducted to assess the collinearity among predictor variables, as well as the direction and strength of their relationships. Specifically, Spearman's rho correlation tests were used to examine associations among categorical ordinal (or ordinal) variables, and chi-squared tests were used for categorical nominal (or nominal) variables. Chi-squared analyses examined the associations between MSI status and type of health services. Multivariate logistic regression with maximum likelihood estimation was used to predict the odds of a college having CHS based on its institutional characteristics.

## Results

The sample of 167 Black-serving MSIs included 71 PBIs and 96 HBCUs (Table 1). Of these, 23 PBIs and 85 HBCUs were 4-year institutions. PBIs were more likely to be 2-year institutions compared with HBCUs. There was no difference in undergraduate enrollment between 4-year HBCUs and PBIs (*p*=0.18). Enrollments ranged from 528–28,990 at 4-year PBIs (μ=3605; *M =* 1713) to 84–11,328 at 4-year HBCUs (μ=2526; *M =* 1812). Among the total sample, HBCUs enrolled an average of 79.98% Black students (range 9–99%) and PBIs enrolled 54.07% Black students on average (range 12–87%). HBCUs reported a greater proportion of Pell grant recipients compared to PBIs (μ=73.93% and 68.48%, respectively).

### Research question 1

Among 4-year institutions, 87.06% of HBCUs and 73.91% of PBIs offered some type of college health services (Table 1). The availability of CHS varied; 64.71% of HBCUs had on-campus providers with prescriptive privileges compared to 39.13% of PBIs (*p*=0.05).

### Research question 2

Because PBIs included a disproportionately large number of 2-year colleges, subsequent analyses were limited to 4-year PBIs and HBCUs. As shown in Table 2, bivariate correlations demonstrated that several variables were positively associated with the presence of on-campus CHS. On-campus CHS was significantly and positively associated with larger undergraduate enrollment (⍴=0.58, *p*<0.001), HBCU designation (⍴=0.22, *p*<0.05), and classification as a 4-year public institution (⍴=0.36, *p*<0.001). Institutions with higher percentages of Pell grant recipients had higher percentages of Black students enrolled (⍴=0.41, *p*<0.001).

**Table 2.** Bivariate analysis of institutional characteristics related to student health centers at Historically Black Colleges and Universities and Predominantly Black Institutions BCU and Predominantly Black Institution

	HC type	NumUG	% Black students	% Pell grant	MSI	Type of institution	Setting
HC type (ordinal)	—						
Number of undergraduates (count)	0.58^a^	—					
% Black students (numeric)	0.02	−0.16	—				
% Pell grant recipients (numeric)	−0.18	−0.44^a^	0.41^a^	—			
MSI category (binary)	0.22^b^	−0.09	0.59^a^	0.32^a^	—		
Type of institution (ordinal)	0.36^a^	0.66^a^	−0.18	−0.24^b^	−0.02	—	
Setting (ordinal)	−0.03	0.07	0.05	−0.09	−0.07	−0.14	*—*

Bivariate analysis was conducted using Spearman's correlation coefficient and chi-square distribution tests as appropriate for each set of variables.

^a^ *p*≤0.001.

^b^ *p*≤0.05.

HC, health center; MSI, Minority Serving Institution; NumUG, number of enrolled undergraduates (count).

A logistic regression model was used to examine the effects of institutional characteristics on the likelihood of having on-campus CHS. The final reduced model is shown in Table 3. Number of undergraduate students, type of institution (public vs. private), and setting were not significant predictors and were not included in the final model. HBCU designation was associated with a greater than fivefold increase in the odds of having an on-campus CHS (OR=5.38, *p*=0.014). One other institutional characteristic was significant. Institutions with a greater percentage of Pell grant recipients were less likely to have CHS (OR=0.96, *p*=0.035) compared with institutions with fewer Pell grant recipients. Percentage of Black students was not significant.

**Table 3.** Results of binary logistic regression 4-year institutions (HC Type: binary—best vs. all others)

	***B***	SE	OR	95% CI	Wald statistic	***p***
% Black students	0.00	0.01	1.00	0.97–1.03	0.04	0.840
% Pell grant recipients^a^	−0.04	0.02	0.96	0.93–1.00	4.47	0.035
MSI Category^a^	1.68	0.69	5.38	1.5–23.16	6.00	0.014

^a^ *p*≤0.05.

*B*, unstandardized beta; CI, confidence interval; OR, odds ratio.

## Discussion

This study generated several noteworthy findings. First, we found that HBCUs were five times more likely than PBIs to have on-campus CHS staffed with prescribers. This suggests that HBCUs are better aligned with ACHA recommendations for high-quality student health services.^64,65^ High quality student health services are associated with improved physical and mental health outcomes among students, including increased academic persistence.^47,52,53^ Although not statistically significant, PBIs were more than twice as likely to have no student health services compared to HBCUs (26% vs. 12%). This lack of statistical significance may have been due to the small sample size.

Students who attend MSIs, such as HBCUs or PBIs, experience unique challenges^46,48^ that impact the accessibility and quality of health services available to them.^10,11,50^ HBCUs and PBIs have an opportunity to reach and educate potential students who may not otherwise have the opportunity for higher education.^3,25,26,28,41,54^ Degree completion can significantly impact an individual's life course by increasing financial stability through improved employment opportunities^25,31,67^; a particularly important effect for historically marginalized populations like Black Americans.^29,31,41^

Regarding academic persistence, HBCUs have higher 150% (6-year) completion rates than PBIs. This disparity may be a reflection of the additional resources made available by federal grant and loan programs to HBCUs,^36^ as well as the mission-driven nature of HBCUs.^27,40^ Higher completion rates may also reflect, at least in part, the greater financial support for student services, including CHS, at HBCUs.^12,27,55^

A second noteworthy study finding was that the presence of a CHS was significantly predicted by the percentage of Pell grant-eligible students. This effect was independent of whether a college was classified as an HBCU or a PBI. Schools with higher percentages of students eligible for Pell grants were significantly less likely to have an on-campus CHS. Pell grant recipients, who have demonstrated exceptional financial need,^20^ are among the most economically disadvantaged and potentially vulnerable.^10,11^ Student health centers, as social and health safety nets for vulnerable student populations,^47,79^ are vital elements of effective student services. Financial stress has been found to be a more significant and intense stressor for Black students compared to their White peers.^11,14–16^

Intersectionality between race and poverty experienced by Black students potentiates poor health outcomes and health inequities that arise out of multiple forms of oppression.^2,13^ Because of the financial barriers associated with accessing health services, many young adults avoid seeking and obtaining health services.^57^ Increased access to prescribing providers in on-campus student health centers may improve health and academic persistence among students at HBCUs and PBIs, with implications for lifelong health and economic benefits.

The resources necessary to provide comprehensive college health services are not equitably distributed. Recognizing that U.S. laws contribute to structural racism, the Biden administration's Equity Action Plan requires that the DOE examines current policies to reduce systemic barriers to health care access.^56^ HBCUs have greater financial resources and mission-related institutional support for student health centers compared with PBIs,^19,21,27,43,44^ including federal funding streams, private donors, and institutional endowments. Compared to HBCUs, PBIs experience inequities in federal funding, as well as private donations and endowments.^6,70^ Colleges with high proportions of Pell grant recipients have student populations that face the greatest barriers to accessing health care. Investments in CHS at PBIs and other institutions serving the most under-resourced and at-risk Black students could advance health equity through the improvement of health outcomes and academic persistence.

### Recommendations

Boland and Gasman note that, for Black students, “HBCUs serve as a direct pipeline to the middle class and must continue to serve this purpose.”^41,p.2^ Improved educational attainment among HBCU students, in turn, yields positive consequences for Black communities.^29^ PBIs similarly provide “access to higher education for many low-income and first generation Black students.”^18,p.3^ Arroyo and Gasman^27^ characterize a “supportive environment” as foundational to HBCUs and core to facilitating student success.^27^ As access to quality health services is positively associated with student health, educational persistence, and degree completion, student health services must be viewed as a core component of a supportive environment that promotes student success at HBCUs and also at PBIs. However, in practice, student services at HBCUs and PBIs are often faced with inadequate resources (financial and human) and lack of public and private funding.^32,37,42,80^

Consistent with ACHA recommendations, HBCUs and PBIs should ensure that students have access to quality health care, ideally through an on-campus CHS. Campus health centers are often overlooked and underfunded among student services. Access to health care is a core social determinant of health and, in turn, student persistence and success. As such, health services must be prioritized in budget line allocations, and extramural funding should be sought to support student health initiatives through governmental sources, philanthropic foundations, and alumni donations.^6,7,40,41,55,69,72,73^ Currently, most donations are earmarked for specific programs.^19,40,43^ Institutions should solicit unrestricted funds that can be applied to general operating costs,^42^ including CHS. An outstanding resource, the UNCF Institute for Capacity Building, partners with HBCUs and PBIs to build capacity, identify revenue sources, and support the adoption of best practices.^81,83^

Finally, equitable public funding for both HBCUs and PBIs would positively enhance the ability of these institutions to meet student needs. Several authors^6,41,72,82^ have asserted that states prioritize funding to PWIs and flagship universities over MSIs. Boland and Gasman recommend that “state governments recognize the efficacy and relevance of HBCUs and their missions.^41,p.11^ Although PBIs may have fewer resources from endowments and alumni donations, they may be eligible to apply to PBI Formula Grants; these have the stated purpose to strengthen eligible institutions' capacity to serve more low- and middle-income Black students and to support Black student persistence in postsecondary education.^43^ However, PBI Formula Grants appear limited to fairly narrowly defined academic initiatives. A more holistic conceptualization of what contributes to student persistence and success would advocate for the use of such funds to expand campus services to promote mental and physical health services for students.

### Limitations

This study was a secondary analysis of data abstracted from multiple sources. Therefore, the study is limited by the size of the existing sample and the available variables.^84^ The sample size of 167 universities/colleges was relatively small and may have limited analytic statistical power. There was also an uneven distribution of institution types between PBIs and HBCUs. Some of the study data were abstracted from college websites; it was assumed that these data were accurate. Finally, other variables of potential interest may not have been available in the dataset.

## Conclusions

Access to health care is an important social determinant that affects health, academic persistence, and degree completion, all of which can impact an individual's life course.^39,59,62,68^ This study found that HBCUs were significantly more likely than PBIs to offer comprehensive on-campus student health services for students. While federal funding supports the development of student health centers at HBCUs, no equivalent funding stream exists for PBIs.^19,21,43,44^ Increased public funding for PBI health centers could improve access to health care and promote health equity. Future research should include measures of student health center funding, assessment of the quality and utilization of student health services, and examination of linkages with student health outcomes.

## Abbreviations Used

ACHA: American College Health Association
CHS: comprehensive health services
CI: confidence interval
DOE: Department of Education
EMT: emergency medical technician
HBCUs: Historically Black Colleges and Universities
HC: health center
IPEDS: Integrated Postsecondary Education Data System
LPN: licensed practical nurse
MSI: Minority Serving Institutions
NCES: National Center for Education Statistics
NumUG: number of enrolled undergraduates
OR: odds ratio
PBI: Predominantly Black Institution
PWI: Predominantly White Institution
RN: registered nurse
UNCF: United Negro College Fund
